# Alpha-Hederin induces incomplete autophagic injury in non-small cell lung cancer by interfering with the lysosomal acidification

**DOI:** 10.1038/s41598-024-63348-6

**Published:** 2024-06-10

**Authors:** Feng Jin, Xiaomin Jiang, Xiaochen Ni, Shilong Yu, Feng Wu, Xinlin Shi, Defang Mao, Haibo Wang, Qingtong Shi, Yanqing Liu, Qin Xu

**Affiliations:** 1 Department of Respiratory Medicine, The Affiliated Hospital of Yangzhou University, Yangzhou University, Yangzhou, 225001 China; 2 Department of Thoracic Surgery, The Affiliated Hospital of Yangzhou University, Yangzhou University, Yangzhou, 225001 China; 3 Institute of Translational Medicine, Medical College, Yangzhou University, Yangzhou, 225001 People’s Republic of China; 4 The Key Laboratory of Syndrome Differentiation and Treatment of Gastric Cancer of the State Administration of Traditional Chinese Medicine, Yangzhou, 225001 China; 5 Yangzhou Hospital of Traditional Chinese Medicine, Yangzhou, 225001 China

**Keywords:** α-Hederin, Incomplete autophagy, Lysosomal acidification, Non-small cell lung cancer, Antitumor, Biochemistry, Cancer, Chemical biology

## Abstract

Lung cancer is the most common oncological disease worldwide, with non-small cell lung cancer accounting for approximately 85% of lung cancer cases. α-Hederin is a monodesmosidic triterpenoid saponin isolated from the leaves of Hedera helix L. or Nigella sativa and has been extensively studied for its antitumor activity against a variety of tumor cells. It has been suggested that α-Hederin is a potential regulator of autophagy and has high promise for application. However, the specific mechanism and characteristics of α-Hederin in regulating autophagy are not well understood. In this study, we confirmed the potential of α-Hederin application in lung cancer treatment and comprehensively explored the mechanism and characteristics of α-Hederin in regulating autophagy in lung cancer cells. Our results suggest that α-Hederin is an incomplete autophagy inducer that targets mTOR to activate the classical autophagic pathway, inhibits lysosomal acidification without significantly affecting the processes of autophagosome transport, lysosome biogenesis, autophagosome and lysosome fusion, and finally leads to impaired autophagic flux and triggers autophagic damage in NSCLC.

## Introduction

Lung cancer is the most common cause of cancer-related deaths in men and women worldwide. Non-small cell lung cancer (NSCLC) is the most common type with high incidence and mortality rates^1^. With an estimated 1.6 million deaths per year, lung cancer is a major global health threat^2^. To date, the main strategies of cancer treatment include surgery, radiotherapy, and chemotherapy. Surgery and radiotherapy are usually used to remove tumor lesions, while chemotherapy is mainly used as postoperative adjuvant therapy in clinical practice because chemotherapeutic drugs can generally spread throughout the body and effectively kill tumor cells^3^. For patients with advanced tumors, there is usually no surgical opportunity to eradicate the tumor, and chemotherapy is the first-line treatment for advanced lung cancer, which can prolong patients' survival and improve their quality of life^4^. Attention has been drawn to autophagy as an important mechanism of chemoresistance in NSCLC, enabling NSCLC cells to increase their survival in the presence of sustained stress^5^. Autophagy is an evolutionarily conserved process in which autophagic vesicles wrap the cytoplasmic components of cells and transport them to lysosomes for degradation^6^. Autophagy plays an important role in maintaining intracellular metabolic homeostasis by removing dysfunctional or unnecessary proteins and damaged or aged organelles to recycle their metabolites, thereby maintaining cell survival and genetic stability^7^. When cells encounter environmental stressors such as pathogenic infections and nutritional deprivation, autophagy provides nutrients and energy necessary for cell survival. Therefore, autophagy is considered as a cytoprotective effect against environmental stress^8,9^. Moreover, autophagy can rapidly promote cellular drug resistance, which severely limits the efficacy of chemotherapeutic agents^6^. In late stages of cancer, tumor cells often experience environmental stresses such as lack of nutrition, hypoxia or chemotherapy-induced cytotoxicity, yet autophagy induces tumor cell survival and drug resistance. Therefore, inhibition of autophagy during tumor progression may inhibit tumor proliferation along with tumor drug resistance.

CQ (Chloroquine) and HCQ (Hydroxychloroquine), as an inhibitor of late autophagy, have been clinically applied in the treatment of malignant tumors. Encouragingly, clinical results confirm that inhibition of autophagy alone or in combination with chemotherapy is a feasible and effective strategy during tumor progression^10–12^. CQ and HCQ lead to increased sensitivity of tumor cells to different chemotherapeutic agents by inhibiting cytoprotective autophagy. Human cervical cancer cells HeLa are resistant to death receptor-induced apoptosis, but after pretreatment with 75 μM CQ, HeLa cells were sensitive to Fas-mediated apoptosis^13^. Not only that, CQ also increased the toxic effect of cisplatin on Hela cells, even though CQ itself was non-toxic to HeLa. This is mainly attributed to the end-stage blockade of autophagy and the accumulation of misfolded intracellular proteins, which ultimately leads to the activation of apoptosis. In HeLa cells, cisplatin induced the accumulation of misfolded proteins but at the same time increased the flux of complete autophagy, which would accelerate the clearance of ubiquitinated proteins to alleviate the stress of endoplasmic reticulum stress^14^.Not only chemotherapeutic agents such as cisplatin and 5-fluorouracil, therapeutic strategies using CQ and HCQ to block autophagy have a significant potentiation effect on both sunitinib, bevacizumab and oxaliplatin drugs^15,16^.

α-Hederin is a monodesmosidic triterpenoid saponin isolated from the leaves of *Hedera helix* L. or *Nigella sativa* and has been extensively studied for its antitumor activity against a variety of tumor cells. α-Hederin has high antitumor activity and is considered as a promising alternative antitumor agent. In colorectal cancer, α-hederin induces autophagic cell death through AMPK/mTOR signaling pathway^17^. However, it has been shown that α-hederin is an autophagy blocker in non-small cell lung cancer. By blocking the cytoplasmic process of ROS scavenging, α-hederin promoted Paclitaxel cytotoxicity by increasing reactive oxygen species accumulation^18^. Currently, it is unclear why the autophagosomes induced by α-hederin cannot complete the full autophagic flux. To explore this question, we conducted the study in order to shed more light on the regulatory role of α-hederin on autophagy.

## Materials and methods

### Reagents and antibodies

The following antibodies were purchased from Cell Signaling Technology (USA): LC3B (Catalogue No.3868S), LAMP1 (Catalogue No.15665S), mTOR (Catalogue No.2983S), SQSTM1/p62 (Catalogue No.23214S), GAPDH (Catalogue No.92310SF). The following reagents were purchased from Beyotime Biotechnology (China): Tubulin-Tracker Green (Catalogue No.C1051S), Cell Counting Kit-8 (Catalogue No.C0037), DAPI Staining Solution (Catalogue No.C1005), Actin-Tracker Red-555 (Catalogue No.C2203S). The following reagents were purchased from Thermo Fisher Scientific (USA): LysoSensor Green DND-189(Catalogue No.L7535), LysoSensor Green DND-189 (Catalogue No.L7535). Matrix (Catalogue No.356234) was purchased from Corning Incorporated. α-Hederin (CAS. 27,013–91-8) was purchased from Shanghai yuanye Bio-Technology Co., Ltd.

### Cell culture

H1299 was purchased from Shanghai Zhong Qiao Xin Zhou Biotechnology Co.,Ltd., and A549 was purchased from Procell Life Science&Technology Co.,Ltd.. All cell cultures were carried out strictly according to the instructions. RPMI1640 was purchased from HyClone Biochemical Products (Beijing) Co., Ltd. and F-12 K was purchased from Procell Life Science&Technology Co.,Ltd.. The cell culture medium was supplemented with 10% fetal bovine serum (Gibico) and no antibiotics were added. The parameters of the incubator were set to 5% CO2, 37 ℃ and saturated humidity.

### CCK-8 assay

CCK-8 assay was used to detect the inhibitory effect of α-Hederin on NSCLC. Cells were inoculated in 96-well plates at 1000 per well and incubated for 16 h waiting for the cells to recover their morphology. After 24 h of incubation given the indicated concentrations of α-Hederin-containing medium, the drug was withdrawn. 200 µl of 10% CCK-8 solution was added and incubated for 1 h in a 37℃ incubator protected from light, followed immediately by measurement of absorbance values at 450 nm. The measured data were subjected to normalised analysis.

### Colony formation assay

Clonogenic assays were used to test the long-term inhibitory effect of α-Hederin on NSCLC. After treating NSCLC with the indicated concentrations of α-Hederin for 24 h, low density single cell suspensions were prepared and inoculated in 6-well plates at 800 cells per well. Thereafter, the medium was changed every 3 days with fresh medium and the culture was terminated when the clonal population of cells in the control group had grown to more than 60 cells per colony. Cells were fixed with 4% paraformaldehyde and stained with 0.1% crystal violet.

### Transwell assay

The Transwell penetration assay was used to test the effect of α-Hederin on the invasive capacity of NSCLC. The substrate was first spread evenly on the inside of the chambers and placed in an incubator for 2 h to solidify. After 24 h of treatment with the indicated concentrations of α-Hederin, NSCLC cell suspensions were prepared and inoculated in the upper part of the chambers 2 × 104 per well. After 24 h, the cells were gently wiped from the upper chamber with a cotton swab and the chambers were fixed in 4% paraformaldehyde for 30 min. After washing off the paraformaldehyde, the chambers were stained in 0.1% crystal violet solution for 30 min. Finally, the chambers were cleaned and photographed.

### Immunofluorescence

Immunofluorescence assays were used to detect the expression and distribution of autophagic vesicles and lysosomes. NSCLC were pre-cultured on cell crawl slices and given the appropriate drug treatment for 24 h. The slides were prepared by simply fixing 4% paraformaldehyde for 30 min and permeabilising 0.1% TritonX-100 for 10 min at room temperature. After washing the crawling slices, they are blocked with 5% BSA-TBST for 2 h at room temperature. As a rule, primary antibodies are incubated overnight at 4℃ and protected from light (usually 14 h) and secondary antibodies are incubated for 1 h at room temperature and protected from light. The nuclei were stained using Dapi and subsequently imaged.

### Immunoblot

Immunoblotting experiments were used to study the effect of α-Hederin on protein expression. After treating NSCLC cells with the indicated drugs for 24 h, RIPA buffer was used to extract total cellular protein, followed by quantification of protein concentration using BCA assay. Protein lysates were mixed proportionally with Loading buffer and denatured at 95℃ for 5 min. This was followed by vertical electrophoresis to spread the proteins of different molecular weights and horizontal electrophoresis to transfer the proteins onto PVDF membranes. The PVDF membranes were placed in 5% skimmed milk for 2 h at room temperature and the residual milk powder was thoroughly washed off. Typically, primary antibodies are incubated overnight at 4℃ and secondary antibodies with horseradish peroxidase are incubated for 1 h at room temperature. Finally the relative expression of the protein is detected in a gel imaging system.

### Transfection

The preparation and packaging of the virus was done by Shanghai Genechem Co.,Ltd. and the cells were transfected strictly according to the instructions. Briefly, cells were inoculated in 6-well plates at a density of 40,000 cells per well and after 24 h of incubation, the virus was added with the infection reagent (supplied by the company). After 16 h of infection, the medium with the virus was removed and the cells were washed 3 times, followed by the addition of normal medium and continued incubation for about 3 days. When the cells could be observed to fluoresce brightly, puromycin (2 μg/ml) was added and incubation continued for 48 h. When all cells are fluorescent, the cell line is considered to be successfully constructed and the cell culture no longer requires puromycin to avoid any effect on the level of cellular autophagy.

### Live cell imaging

The RFP-GFP-LC3B system is required to detect the localisation and expression of LC3B protein inside live cells. Cells are pre-incubated on surface-treated coverslips and given different drug treatments for 24 h. When it was necessary to observe fluorescent expression inside the cells, the coverslips were attached to slides using a little complete medium and observed directly using a confocal microscope. The whole process was allowed to last no longer than 30 min from the time the coverslip left the culture dish, otherwise the autophagy level of the cells would be affected.

### Lysosomal imaging

To detect the relative number of lysosomes, we used a fluorescent reagent to label lysosomes. lysoTracker Green DND-26 is a fluorescent dye that selectively stains the acidic compartment in living cells. After 24 h of drug treatment, we fluorescently stained NSCLC cells (50 nM, 37 °C, 30 min) and completed the assay using flow cytometry.

### Lysosomal pH detection

Detection of lysosomal pH using LysoSensor Green DND-189. The probe stains acidotropically, accumulates in acidic organelles by protonation and its fluorescence intensity increases progressively with the degree of acidification of the organelle. As the number of lysosomes changed significantly under different drug treatments, the fluorescence intensity was examined in this study using fluorescence microscopy. After 24 h of drug treatment, NSCLC cells were stained (1 μM, 37 °C, 60 min). After staining, residual reagents were thoroughly washed and imaged under a fluorescence microscope. The average fluorescence intensity of the images was measured using imagej software.

### Data analysis and statistics

SPSS 25.0 was used for statistical analysis. Differences between two groups were tested using t-tests, and differences between more than three groups were tested using one-way ANOVA. The differences were considered to be statistically significant when * *P* < 0.05, **P < 0.01, and ***P < 0.001.

## Results

### α-Hederin inhibits the cellular activity of NSCLC

**Figure 1:**
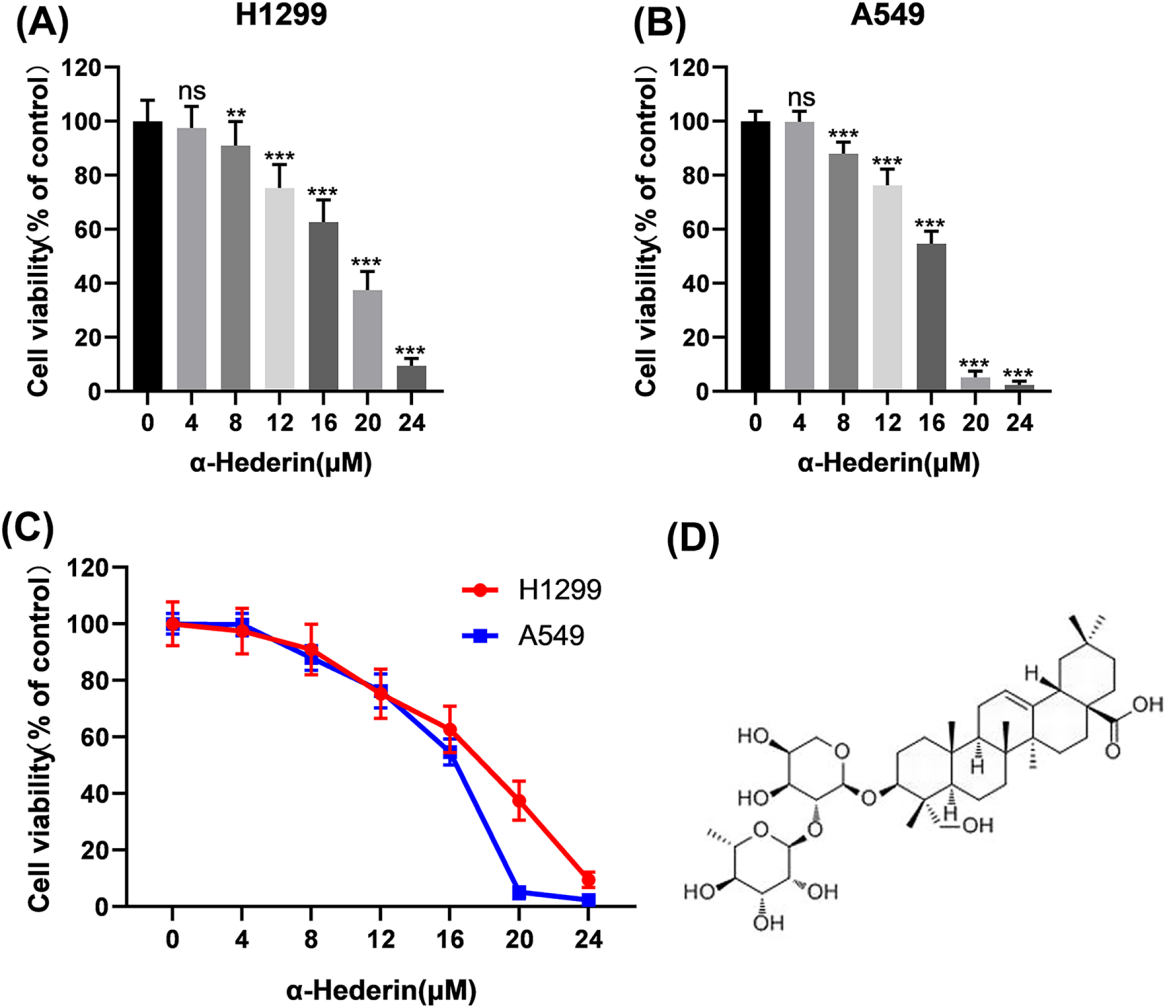
α-Hederin inhibits the cellular activity of NSCLC. (**A**–**C**)CCK-8 assay to detect the cytotoxicity of α-Hederin against H1299 and A549. (**D**) Structural formula of α-Hederin. ns, *P* > 0.05; *, *P* < 0.05; **, *P* < 0.01; ***, *P* < 0.001 vs. Ctrl (Control).

α-Hederin is a monodesmosidic triterpenoid saponin isolated from the leaves of *Hedera helix* L. or *Nigella sativa*, its chemical formula is shown in the Fig. 1D. CCK-8 assays confirmed the cytotoxic effect of α-Hederin on H1299 and A549 (Fig. 1A-C). The cell viability and drug concentration were fitted into the concentration–response curve, and the half inhibitory concentration(IC_50_) of α-Hederin for H1299 and A549 cell lines was calculated to be 23.58μΜ and 14.22 μM, respectively. Therefore, for the next experiments, we used three concentrations of 4 μM, 8 μM, and 16 μM.

### α-Hederin inhibits NSCLC clone formation

**Figure 2:**
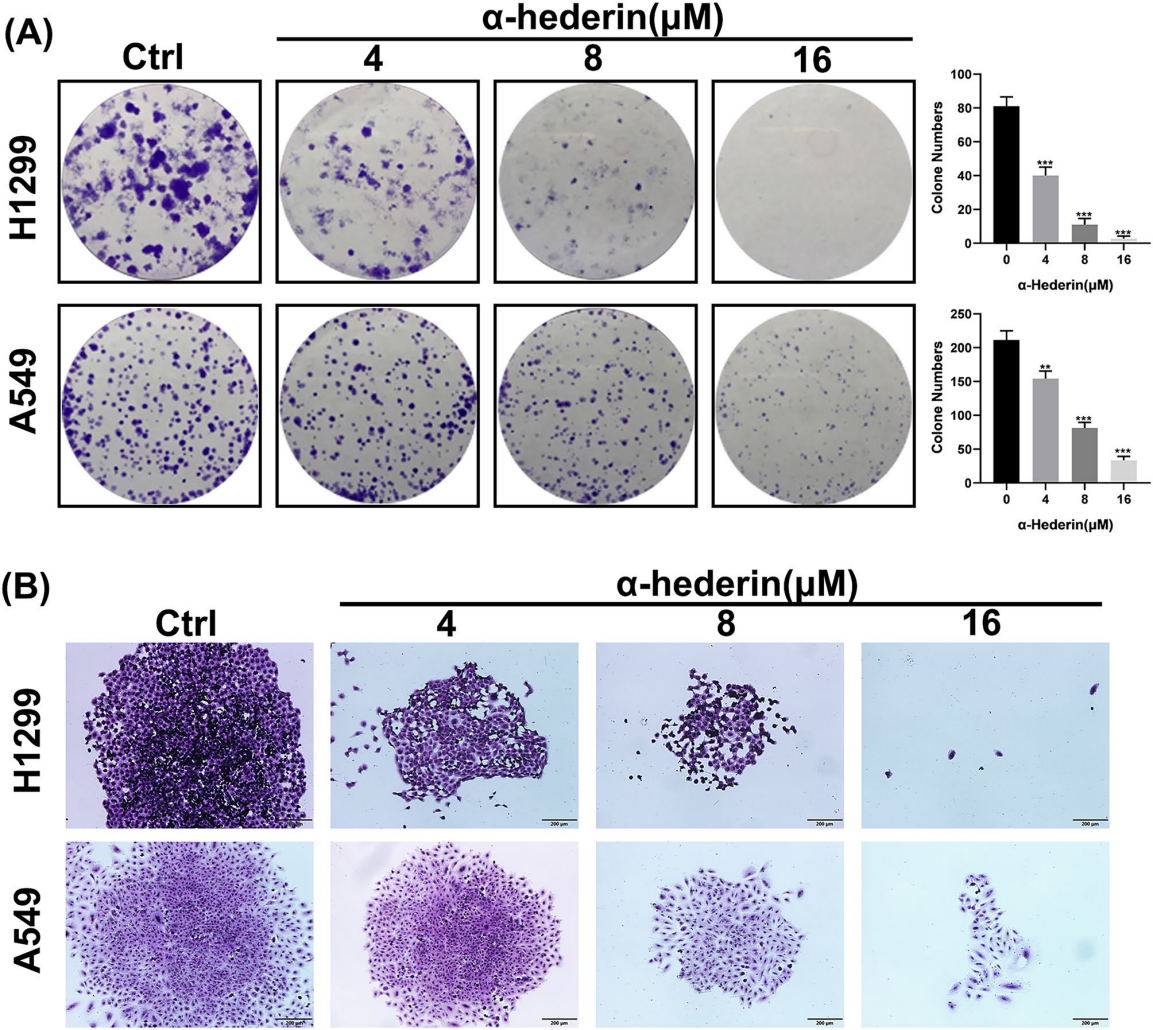
α-Hederin inhibits NSCLC clone formation. (**A**) Clone formation assay was used to detect the long-term growth effects of α-Hederin on H1299 and A549. *, *P* < 0.05; **, *P* < 0.01; ***, *P* < 0.001 vs. Ctrl (Control). (**B**) Representative images of individual clones. Scale bar 200 μm.

To test the long-term inhibitory effect of α-Hederin on NSCLC, we performed a clone-formation assay. The results showed that α-Hederin significantly reduced the number of clone formation in NSCLC and reduced the number of cells in each colony (Fig. 2A-B). These results suggest that α-Hederin can inhibit the survival and proliferation ability of NSCLC in the long term.

### α-Hederin inhibits the invasion ability of NSCLC

**Figure 3:**
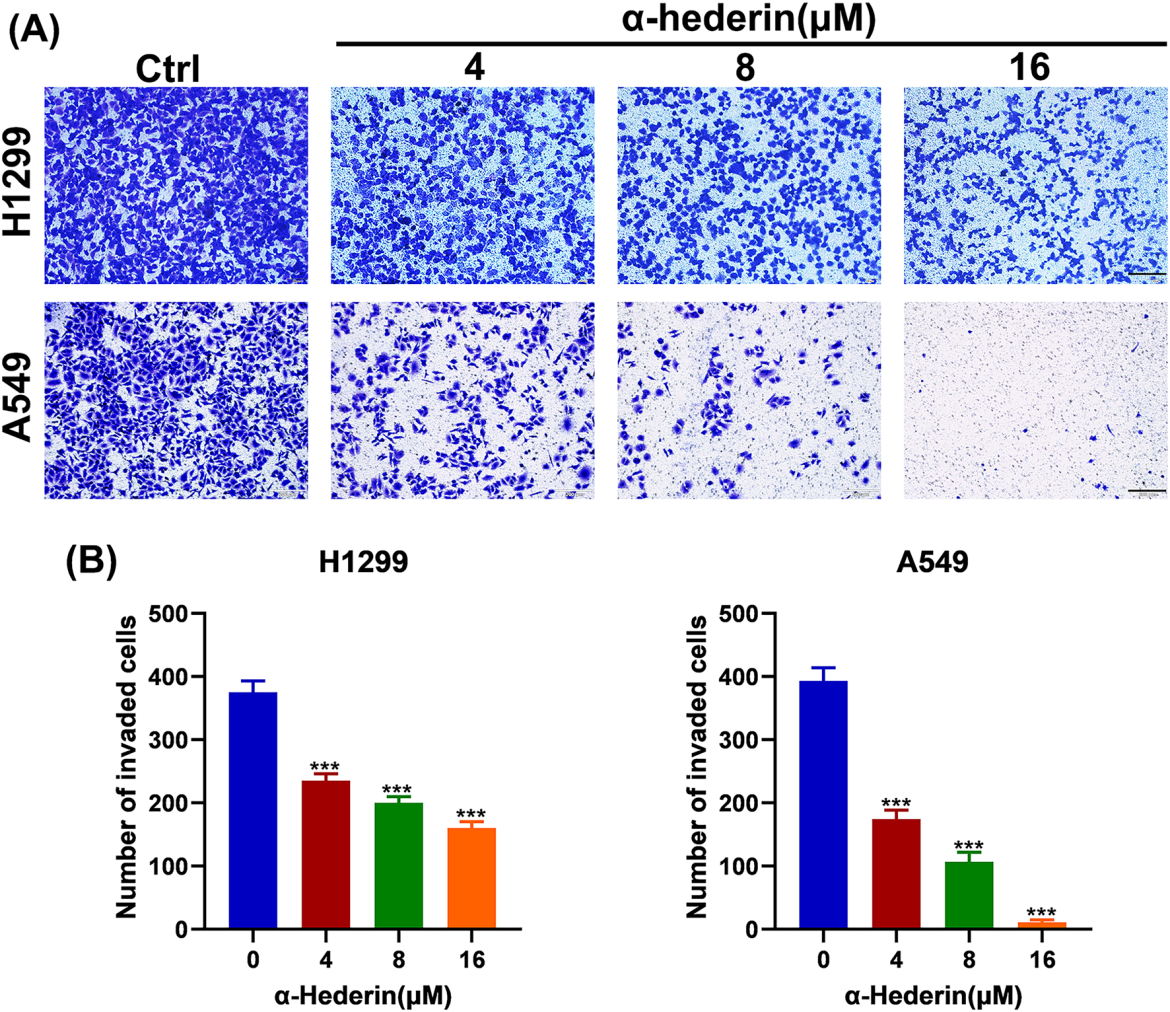
α-Hederin inhibits the invasion ability of NSCLC. (**A**) Transwell chambers were used to detect the effect of α-Hederin on the invasive ability of H1299 and A549. Scale bar 200 μm. (**B**) Statistical analysis of the number of cells cast on the filter membrane. *, *P* < 0.05; **, *P* < 0.01; ***, *P* < 0.001 vs. Ctrl (Control).

We carried out Transwell assay to confirmed that α-Hederin has any effect on the invasive ability of NSCLC. The results showed that α-Hederin significantly inhibited the invasive ability of H1299 and A549 in a dose-dependent manner (Fig. 3A-B). After treating H1299 cells with α-Hederin for 24 h, the average number of cells penetrating the filter membrane decreased from 375.3 in the control group to 235.3, 200.7, and 159.3, respectively. Similarly, after treating A549 cells with α-Hederin for 24 h, the average number of cells penetrating the filter membrane decreased from 194.3 in the control group to 174.3, 107, and 11.7, respectively. Notably, α-Hederin at a concentration of 4 μM significantly reduced the migratory ability of both H1299 and A549 cells, which is a non-cytotoxic and inhibitory concentration. These results suggest that α-Hederin inhibits the invasive ability of NSCLC.

### α-Hederin inhibits cytoskeletal remodeling in NSCLC

**Figure 4:**
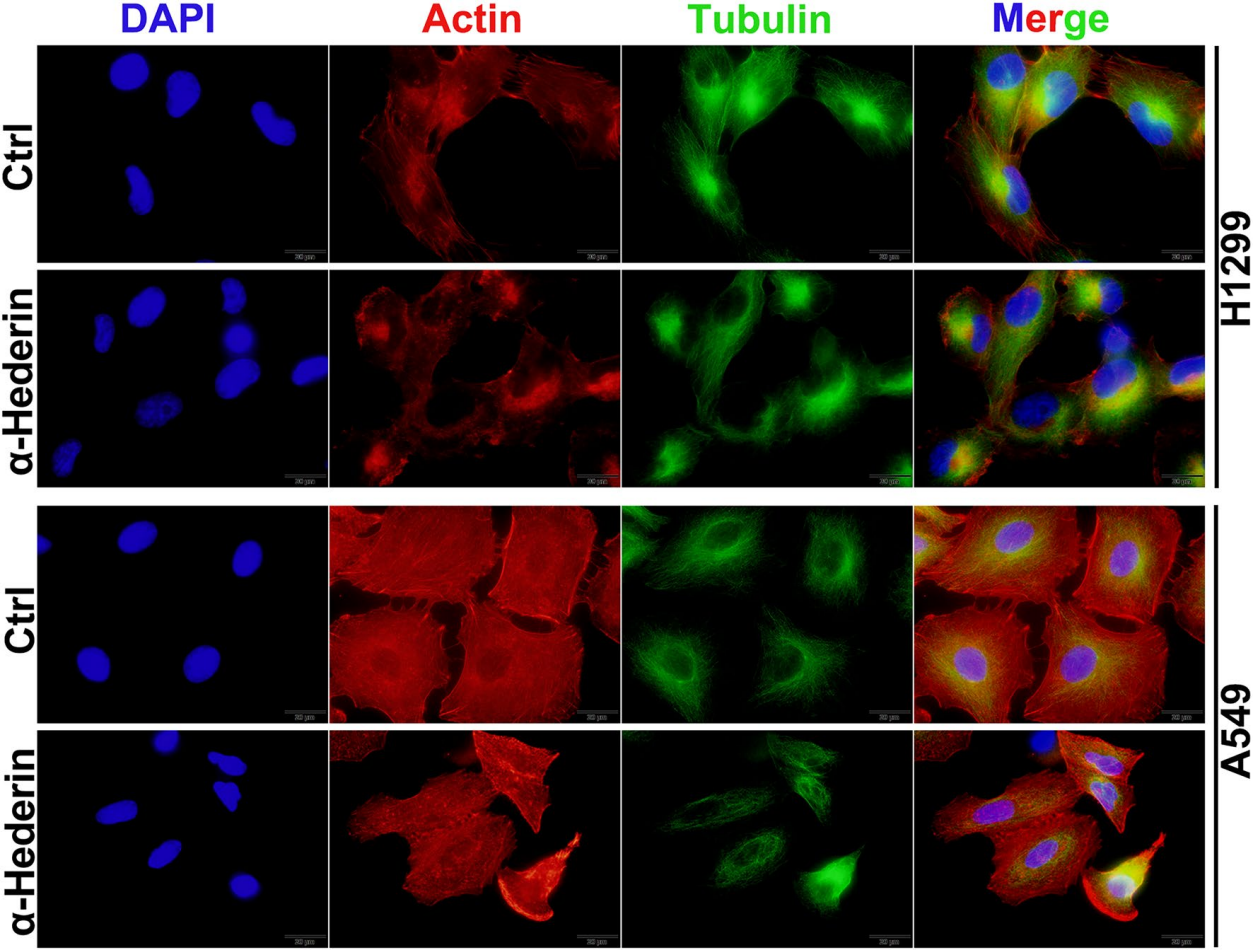
α-Hederin inhibits cytoskeletal remodeling in NSCLC. The intracellular microfilament and microtubule structures were observed by immunofluorescence after α-Hederin(16 μM) treatment of H1299 and A549 for 24 h. Scale bar 20 μm.

Cell invasion and migration must undergo strong changes in the cytoskeleton, driving cells forward through constant dissolution and remodelling of the cytoskeleton. Next, we stained Actin and Tubulin in NSCLC. The results show that after α-Hederin(16 μM) treatment, Actin no longer aggregates into filaments and is enriched in the cell membrane, but is dispersed in the cytoplasm in a punctate manner (Fig. 4), a hallmark change of cytoskeletal remodelling inhibition. At the same time, our results showed that α-Hederin had no effect on the Tubulin structure of NSCLC. These results suggest that α-Hederin inhibits cytoskeletal remodelling by inhibiting the aggregation of Actin.

### α-Hederin activates incomplete autophagy in NSCLC by inhibiting mTOR

**Figure 5:**
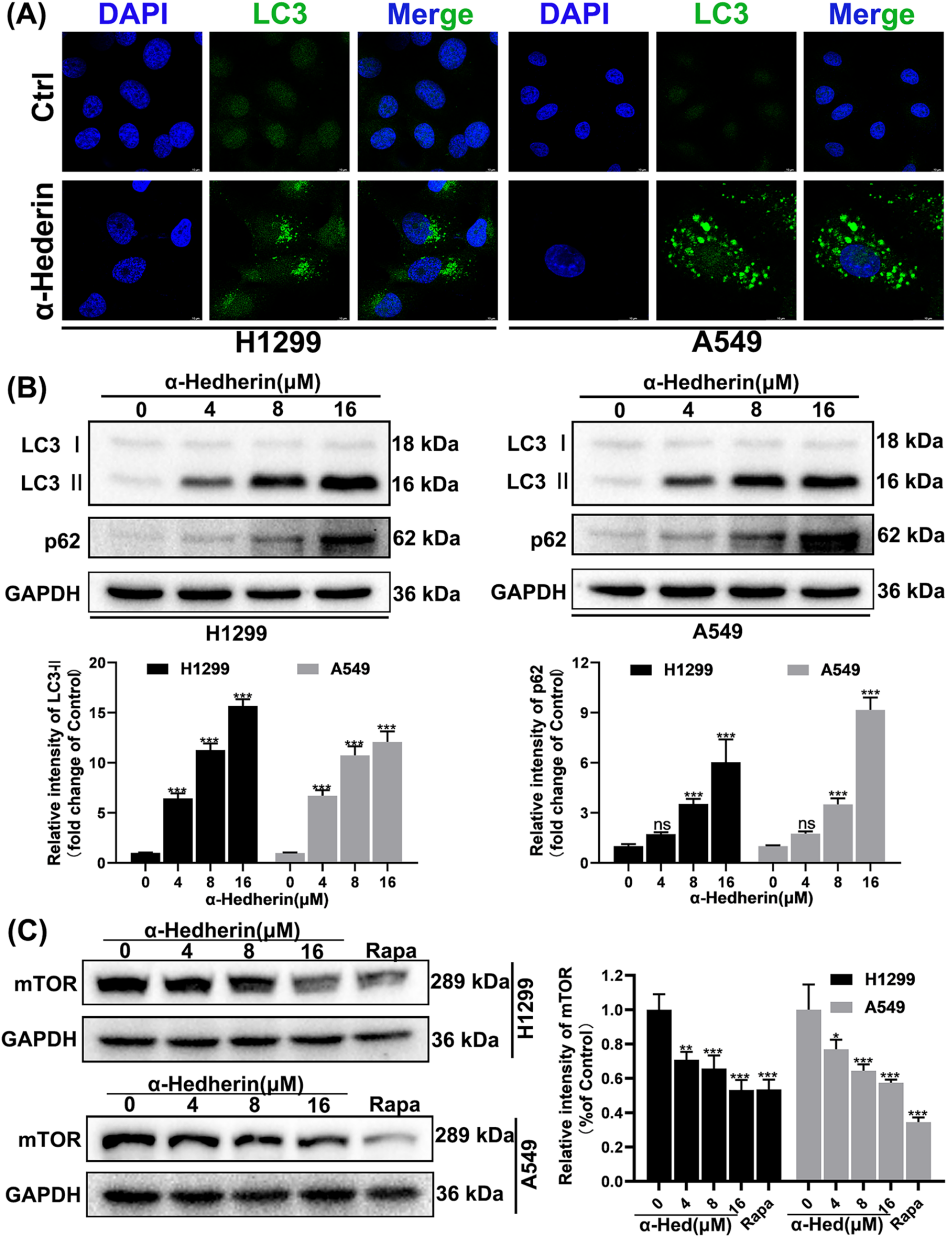
α-Hederin activates incomplete autophagy in NSCLC by inhibiting mTOR. (**A**) α-Hederin(16 μM) treatment of H1299 and A549 for 24 h followed by immunofluorescence assay to label LC3 protein. Scale bar 20 μm. (**B**) α-Hederin(16 μM) treatment of H1299 and A549 for 24 h followed by Western blotting to detect LC3 and p62 protein expression, with GAPDH as an internal reference. (**C**) α-Hederin or Rapamycin(100 nM) treatment of H1299 and A549 for 24 h followed by Western blotting to detect mTOR protein expression, with GAPDH as an internal reference. *, *P* < 0.05; **, *P* < 0.01; ***, *P* < 0.001 vs. Ctrl (Control).

Previous studies have established that α-Hederin causes the accumulation of autophagic vesicles. To further elucidate the effect of α-Hederin on autophagy in NSCLC, we investigated changes in the expression of autophagy-related proteins. lC3 protein, a marker of mature autophagic vesicles, accumulated significantly in α-Hederin-treated NSCLC and aggregated into puncta (Fig. 5A-B). p62, a ubiquitin-binding protein, in α-Hederin-treated(16 μM) NSCLC significant accumulation indicates that autophagic vesicles are not ultimately degraded, which is usually a sign of incomplete autophagy. Chloroquine(20 μM), a blocker of end-stage autophagy, was used as a positive control. In parallel, we found a substantial downregulation of mTOR (Fig. 5C), which is thought to be an activation trigger for incomplete autophagy^19^. Rapamycin(100 nM) was used as a positive control, which is an inhibitor of mTOR. These results suggest that α-Hederin initiates incomplete autophagy in NSCLC by inhibiting mTOR.

### α-Hederin prevents autophagic vesicles from being degraded by lysosomes

**Figure 6:**
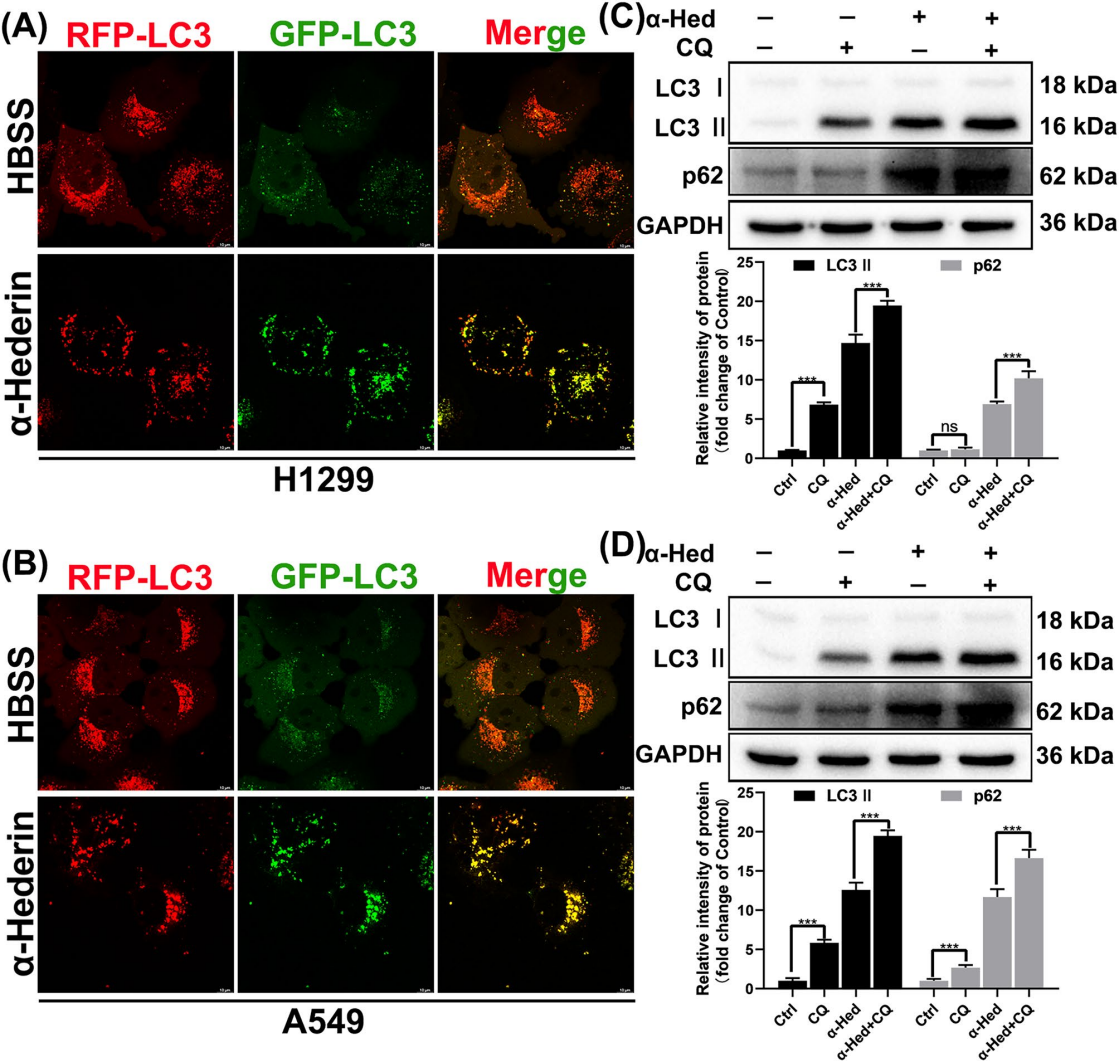
α-Hederin prevents autophagic vesicles from being degraded by lysosomes. (**A**-**B**)After tagging RFP and GFP to LC3 protein, imaging was performed using laser confocal microscopy. HBSS is used to simulate an environment with nutrient deficiency, the concentration of α-Hederin was 16 μM. Scale bar 10 μm. (**C**-**D**) α-Hederin(16 μM) or Chloroquine(20 μM) treatment of H1299 and A549 for 24 h followed by Western blotting to detect LC3 and p62 protein expression, with GAPDH as an internal reference. *, *P* < 0.05; **, *P* < 0.01; ***, *P* < 0.001.

To further demonstrate that α-Hederin-induced autophagy does not complete the final degradation process, we inserted LC3 protein sequences with RFP and GFP into NSCLC. When the LC3 protein is transported into the lysosome, the fluorescence of the GFP protein is quenched due to the low pH value, whereas RFP shows acid resistance, so that it is possible to determine whether the autophagic vesicles are digested by the lysosome by comparing the fluorescence intensity of the RFP and GFP proteins. HBSS is a well-known inducer of autophagy and in the presence of nutrient deficiency, cells initiate the autophagic process and transport the contents to the lysosomes for digestion. We can see that HBSS induces the accumulation of autophagic vesicles, on which the green fluorescent protein is clearly quenched, indicating that the autophagic vesicles fuse with the lysosomes and are digested. In contrast, the α-Hederin-induced(16 μM) autophagic vesicles with the green fluorescent protein on them still emitted bright light (Fig. 6A-B). Compared to the sole use of chloroquine(20 μM), α-Hederin induced the accumulation of more LC3B and p62 proteins. When α-Hederin(16 μM) was used in combination with chloroquine(20 μM), a higher level of LC3B and p62 proteins was observed (Fig. 6C-D). This indicates that α-Hederin blocked the digestion of autophagosomes while promoting their formation, and only a small portion of autophagosomes can bind to lysosomes and be digested. These results suggest that α-Hederin prevented the autophagic vesicles from being degraded by lysosomes.

### α-Hederin did not prevent fusion of autophagic vesicles with lysosomes

**Figure 7:**
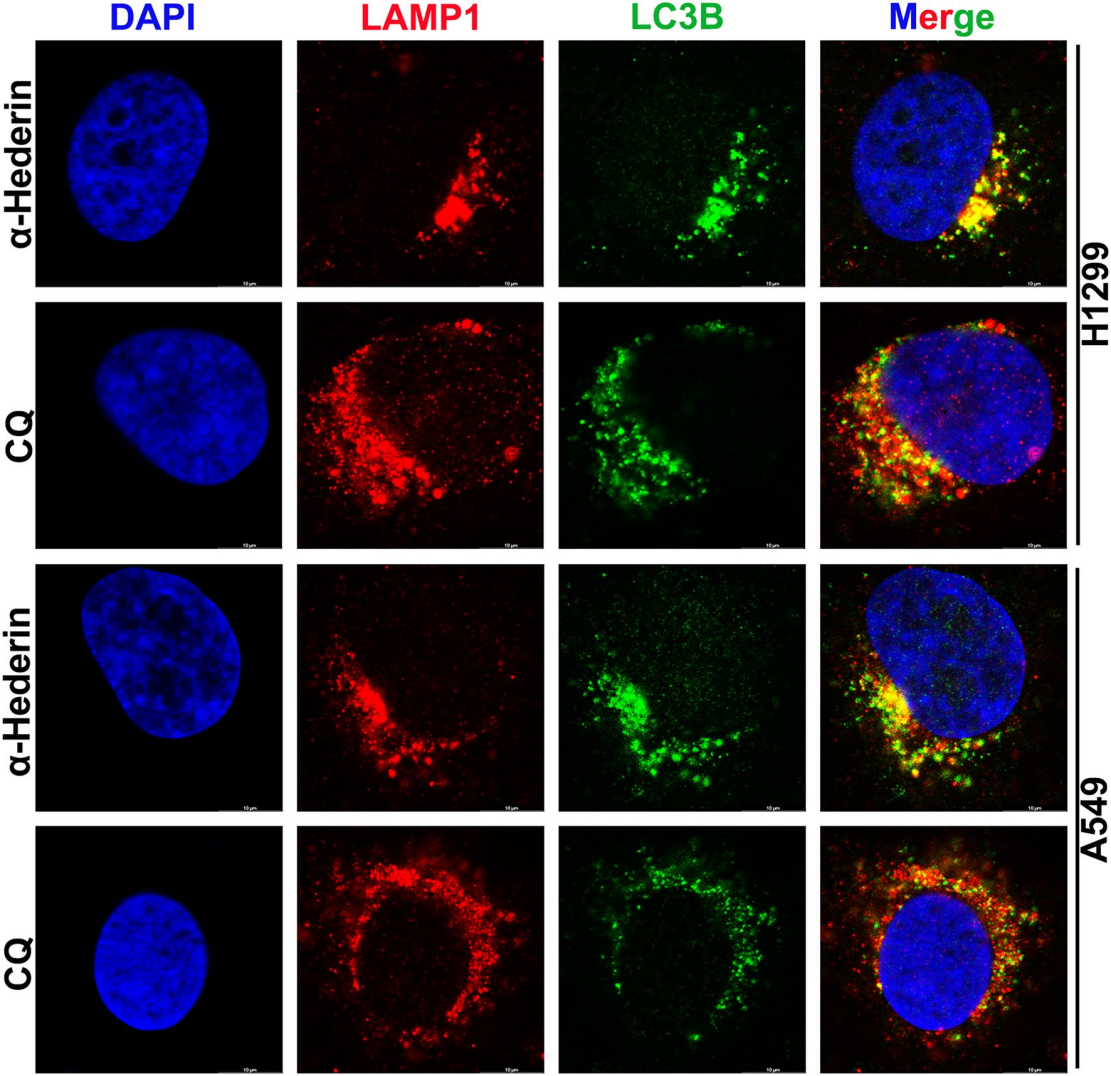
α-Hederin did not prevent fusion of autophagic vesicles with lysosomes. After α-Hederin(16 μM) or Chloroquine(20 μM) treatment of H1299 and A549 for 24 h, labeling LAMP1 and LC3B proteins by immunofluorescence assay. Imaging was performed under a laser confocal microscope. Scale bar 10 μm.

We suggest that there may be two reasons why GFP is not quenched; autophagosomes are not fused to lysosomes, or lysosomes have lost their ability to degrade. We next investigated the relative position of lysosomes to autophagosomes using immunofluorescence co-localisation techniques, with LAMP1 as the lysosomal marker. From the results, it appeared that α-Hederin-induced(16 μM) autophagosomes largely co-localised with lysosomes (Fig. 7). CQ, a drug that blocks the fusion of autophagosomes with lysosomes, was used as a positive control. Thus, these results suggest that α-Hederin does not appear to block the fusion process of autophagic vesicles with lysosomes.

### α-Hederin causes lysosomal accumulation and impaired acidification in NSCLC

**Figure 8:**
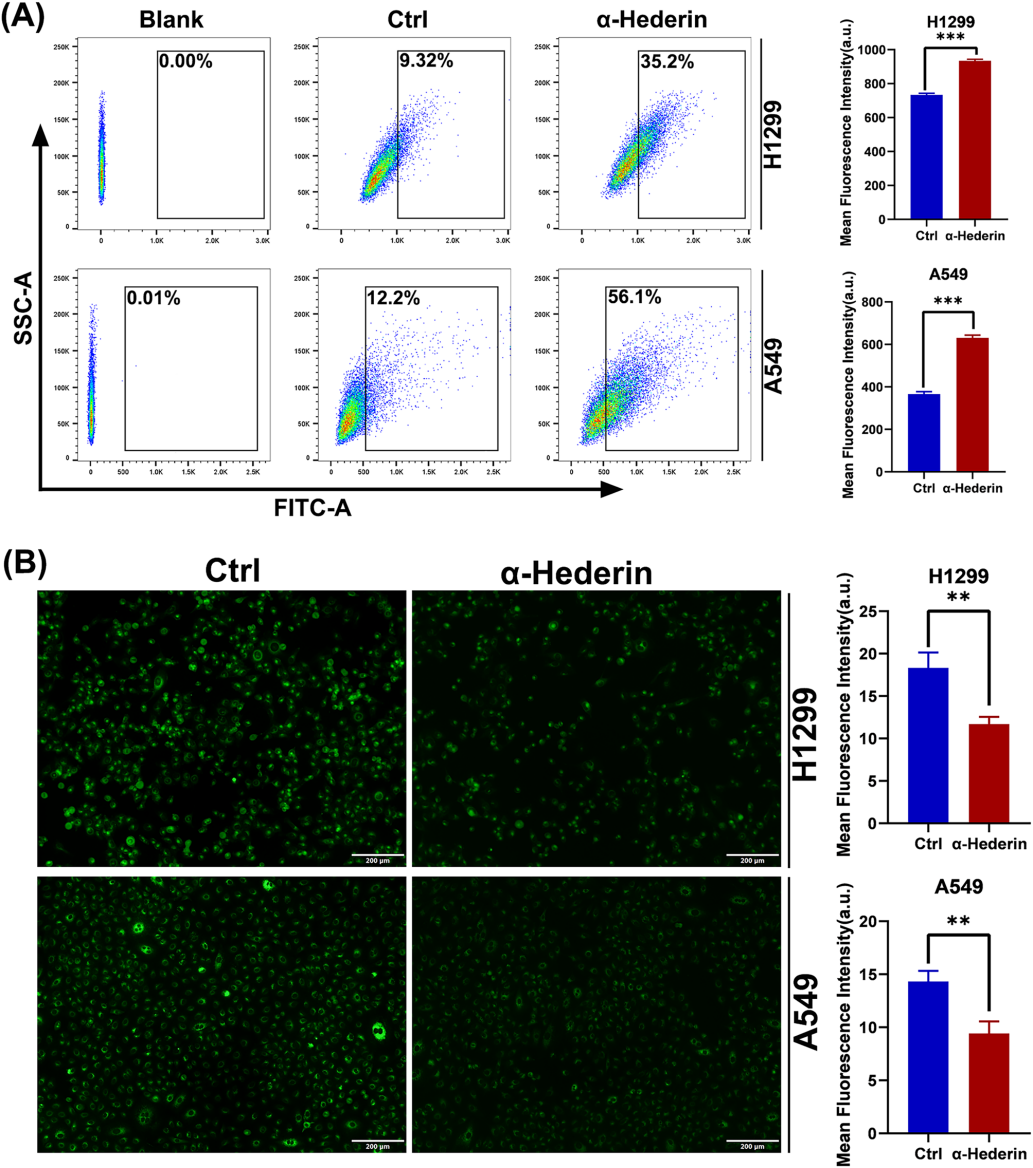
α-Hederin causes lysosomal accumulation and impaired acidification in NSCLC. (**A**) Flow cytometry analysis was performed after lysosomal labeling using lysoTracker Green DND-26, the concentration of α-Hederin was 16 μM. (**B**) Fluorescence imaging was performed after labeling H1299 and A549 using LysoSensor Green DND-189, the concentration of α-Hederin was 16 μM. Scale bar 200 μm. *, *P* < 0.05; **, *P* < 0.01; ***, *P* < 0.001 vs. Ctrl (Control).

Lysosomal acidification is an important prerequisite for its degradation function, and we next examined the degree of lysosomal acidification. LysoTracker Green DND-26 was used to label lysosomes and fluorescence quantification was performed using flow cytometry. The results showed a large accumulation of lysosomes in NSCLC cells after treatment with α-Hederin(16 μM) (Fig. 8A). Therefore, to avoid interference of the results by changes in the number of lysosomes, we performed fluorescence quantification of the lysosomal PH probe using fluorescence microscopy (Fig. 8B). The results showed that α-Hederin significantly reduced the acidification of lysosomes in NSCLC. These results suggest that α-Hederin disrupts the lysosomal acidification capacity of NSCLC.

## Discussion

According to the American Cancer Society, 238,340 new cases (117,550 in men and 120,790 in women) of lung cancer are expected to occur in 2023^20^. Although the 5-year survival rate for lung cancer exceeds 20% for the first time in 2022, we still have a very difficult road to travel after a simple celebration. Compared to synthetic drugs, natural compounds have been selected by nature over a long period of time and present a richer biological function and higher biological activity^21^. α-Hederin is a monodesmosidic triterpenoid saponin isolated from the leaves of Hedera helix L. or Nigella sativa, which has been shown to have excellent anti-tumor activity^22^. The results of CCK-8 assay, clone formation assay and Transwell assay showed that α-Hederin significantly inhibited the proliferative activity and invasive potential of NSCLC and has potential to be used in lung cancer treatment.

Autophagy is an evolutionarily well-conserved cytoplasmic process that forms new metabolic substrates by transporting damaged organelles and pathogens from the cytoplasm to lysosomes where they are eventually digested by hydrolases^23–27^. This is an important survival strategy and the autophagic process plays a crucial role in maintaining intracellular homeostasis when cells are faced with nutritional and environmental stresses[28; 29]. In recent years, an aberrant type of autophagy has been identified, referred to as incomplete autophagy^30^. Like complete autophagy, incomplete autophagy forms autophagosomes through the classical autophagic pathway; however, autophagosomes formed by incomplete autophagy cannot be digested by hydrolytic enzymes and thus accumulate inside the cell, ultimately leading to autophagy-induced cell death^31–34^. In contrast to the general double-edged effect of autophagy that promotes cell survival or death, incomplete autophagy plays a key role in disrupting cellular homeostasis and only promotes cell death. Therefore, targeting incomplete autophagy may contribute to the development of a new generation of therapeutic agents for a variety of human diseases^19^. The current clinical use of chloroquine acting autophagy inhibitors for the treatment of oncological diseases has yielded good results, confirming that blocking autophagy is a promising therapeutic strategy. Although there is no shortage of effective autophagy inhibitors, however, there are very few specific inhibitors that can actually be used for disease treatment. At the same time, targeting autophagy often produces different outcomes of inhibiting and promoting tumor survival, which makes the role of targeted autophagy in drug resistance even more elusive^5^. Therefore, compounds capable of inducing incomplete autophagy are of good developmental value. Our results show that α-Hederin is able to activate the classical autophagy pathway by inhibiting mTOR (Fig. 5), which is consistent with previous studies^17^. Compared to treatment with chloroquine alone, α-Hederin induced more accumulation of LC3B and p62, further confirming the activation of autophagy by α-Hederin(Fig. 6). A large accumulation of L3CII and p62(Fig. 5) suggests that α-Hederin induces incomplete autophagy, because p62 is degraded with the fusion of autophagosomes with lysosomes^35^. Meanwhile, the results demonstrated by the LC3-GFP-RFP reporter system(Fig. 6) are the strongest evidence that α-Hederin induces incomplete autophagy. Regardless of the reason, the autophagosomes did not eventually enter into the acidic environment to be degraded, as the green fluorescent protein GFP is unable to resist acidic conditions. Therefore, we define α-Hederin more accurately as an incomplete autophagy inducer rather than an autophagy inhibitor.

The primary condition for autophagy to occur is to trigger the accumulation of autophagosomes, of which the degradation of mTOR is one of the most common factors^19^. When autophagosomes are formed, the process of autophagosome transport involving the cytoskeleton is extremely important, assisted by kinesin, dynein, and motor proteins, and eventually guided by microtubules to complete the fusion process with lysosomes^36^. Disruption of any of these steps may lead to the occurrence of incomplete autophagy, such as cytoskeletal disruption, impaired lysosomal biogenesis process, and lysosomal dysfunction^19^. Our results suggest that α-Hederin is able to inhibit the remodeling of Actin(Fig. 4), which may be an important mechanism for its inhibition of NSCLC motility(Fig. 3)^37^. Compounds like chloroquine, bafA1 and others that can affect lysosomal acidification can influence the fusion of autophagosomes and lysosomes, but it has been shown that lysosomal acidification is not a prerequisite for the fusion of autophagosomes and lysosomes^38^. Consistent with previous studies^18^, this research confirms that α-Hederin induces lysosomal acidification defects. Our study also found that α-Hederin does not actually impede the fusion of autophagosomes with lysosomes, as can be observed from immunocolocalization images(Fig. 8). This is consistent with our observation of the integrity of microtubules(Fig. 4), because the transportation of autophagosome to lysosome requires the assistance of microtubules. These results suggest that impaired lysosomal acidification may be an important factor in the incomplete autophagy induced by α-Hederin, rather than being related to the transport of autophagosomes, lysosomal biogenesis, or fusion between autophagosomes and lysosomes.

Overall, this study comprehensively explored the causes and mechanisms of incomplete autophagy induced by α-Hederin in terms of autophagosome genesis, delivery, fusion with lysosomes, and lysosomal acidification. Compared to previous studies, this study focused more on the properties of α- hederin-induced autophagy in addition to validating α-Hederin's potential for use in the treatment of lung cancer. This study suggests that impaired lysosomal acidification may be the most important cause of α-Hederin-induced incomplete autophagy.

## Human and animal Research

The research on plants and their compounds in this study complies with relevant institutional, national, and international guidelines and legislation. The sources of compounds and plants can be traced.

### Supplementary Information

Supplementary Figures.

## Data Availability

All data were obtained from Yangzhou University and affiliated institutions, and the corresponding author can provide all original content. We declare that no data were derived from third parties, and no paper mill was used.
