# Supplementary material for: Alpha-Hederin induces incomplete autophagic injury in non-small cell lung cancer by interfering with the lysosomal acidification
Source: Sci Rep. 2024 Jun 10;14:13258. doi: 10.1038/s41598-024-63348-6 (PMC11164981; doi:10.1038/s41598-024-63348-6)
Supplement: Supplementary file 1 — Supplementary Figures. [file 41598_2024_63348_MOESM1_ESM.docx]

Here are all the Western Blot images mentioned in the article, including two imaging modes and merged images for each image. The first one is the chemiluminescence mode, the second one is the bright field mode, and the last one is the merged image.

Chemiluminescence bright field merge


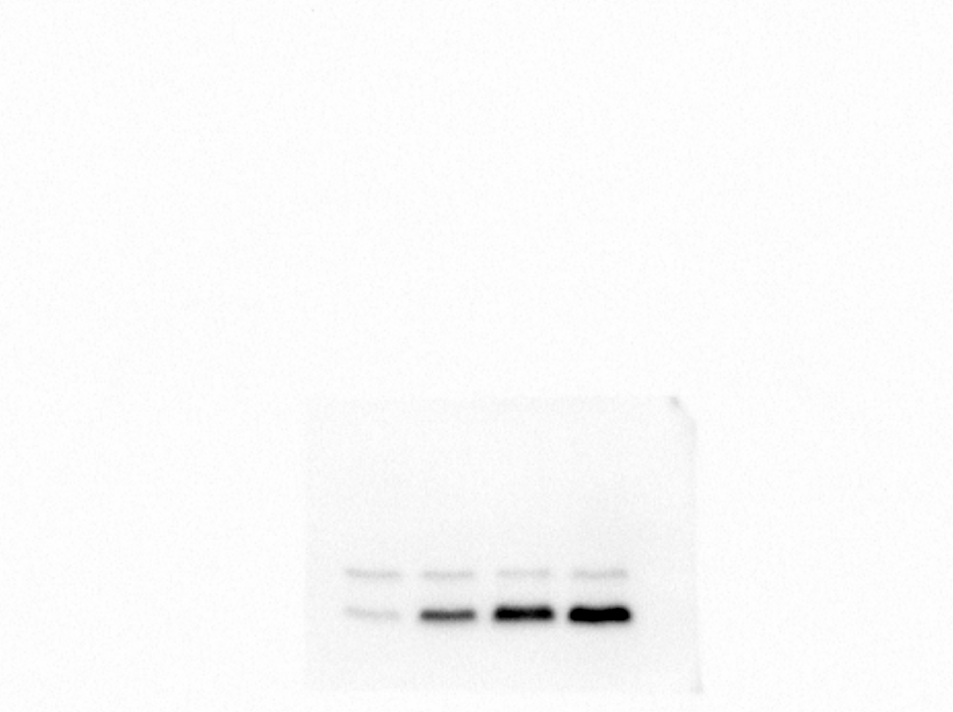

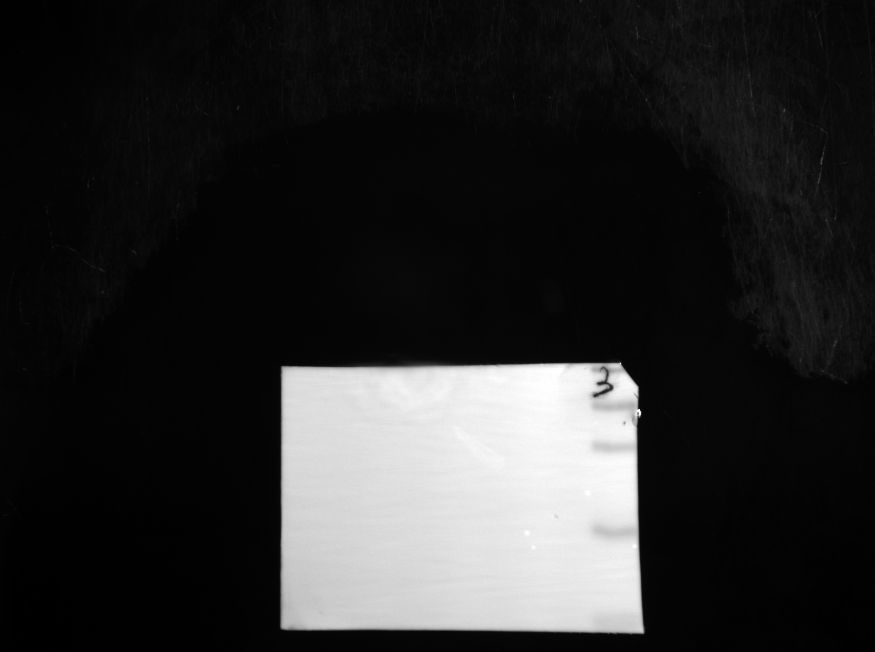

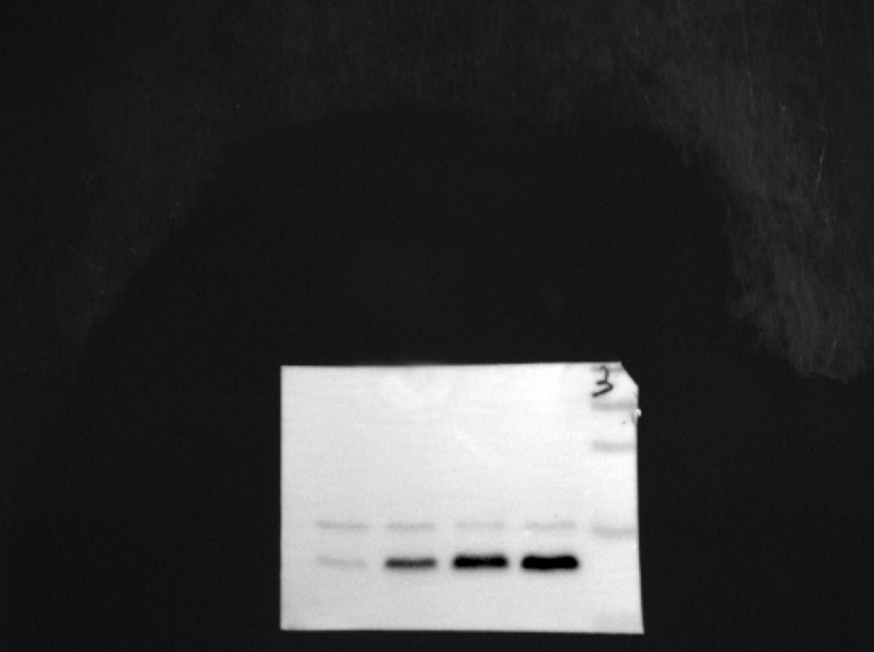


Figure 5. B The LC3 protein band of H1299 cells.


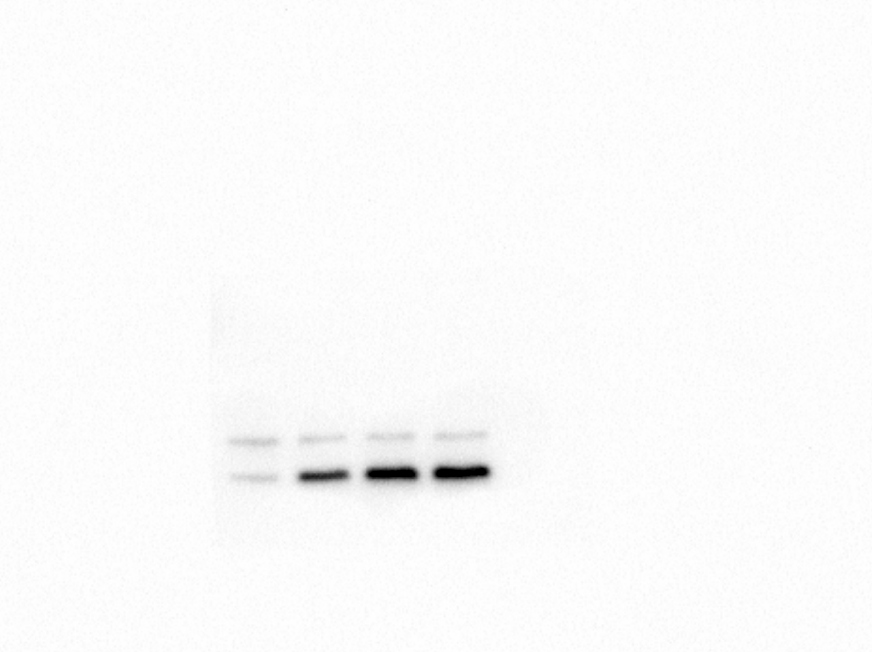

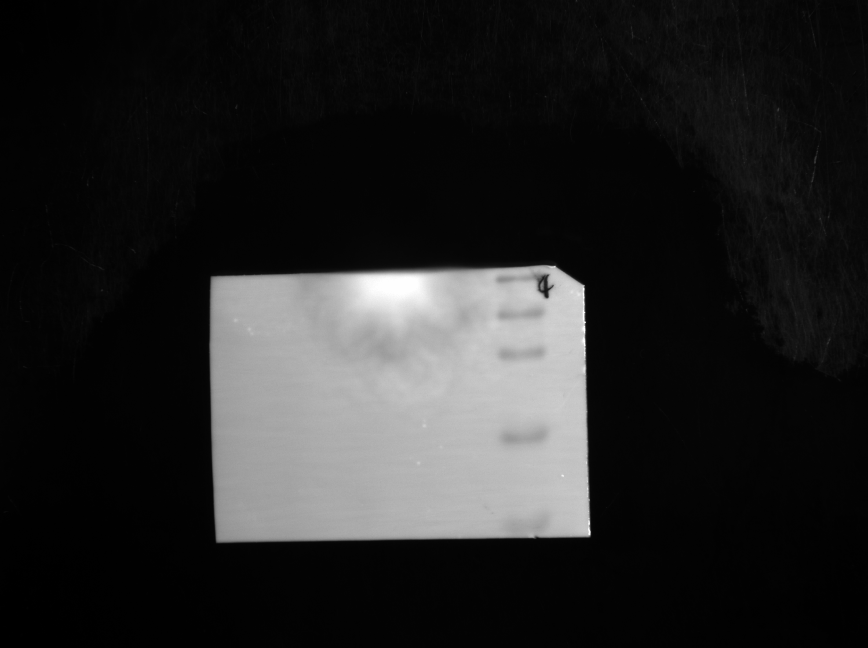

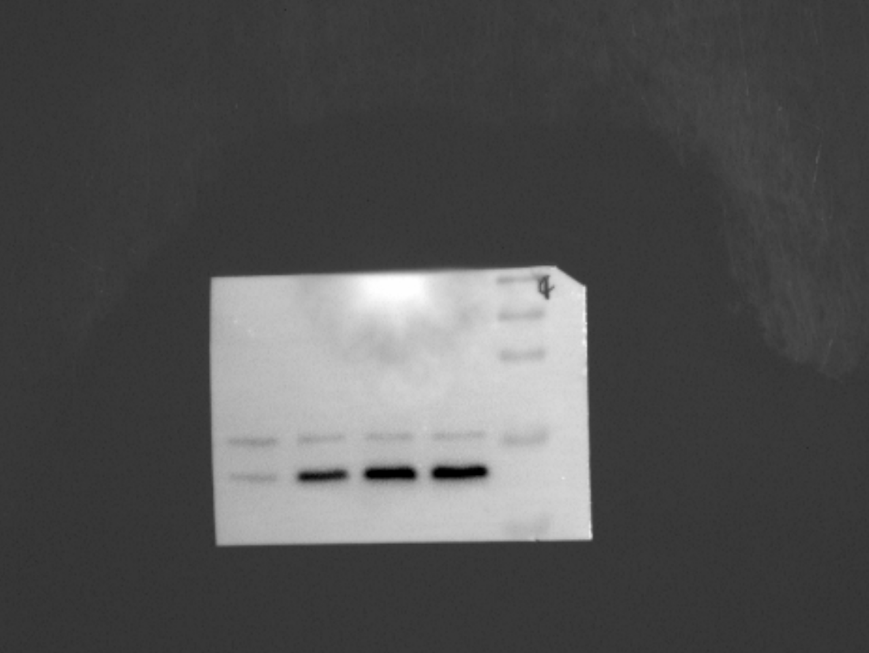


Figure 5. B The LC3 protein band of A549 cells.


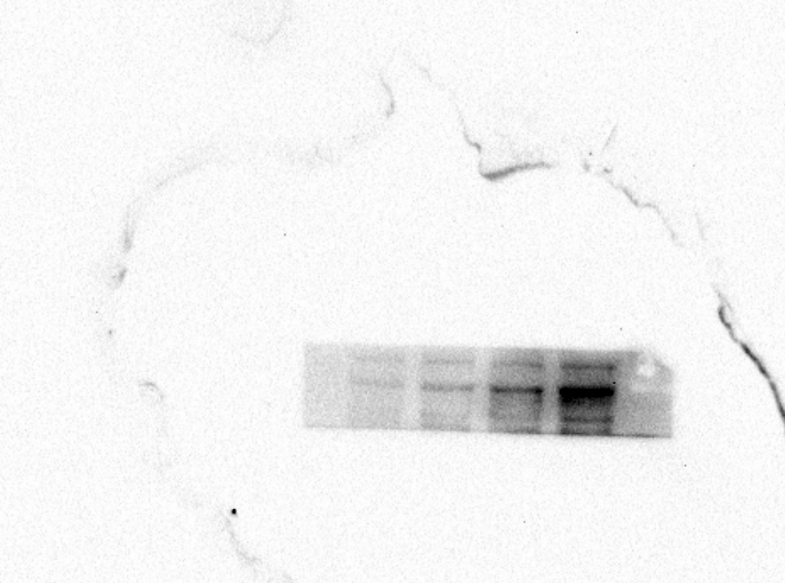

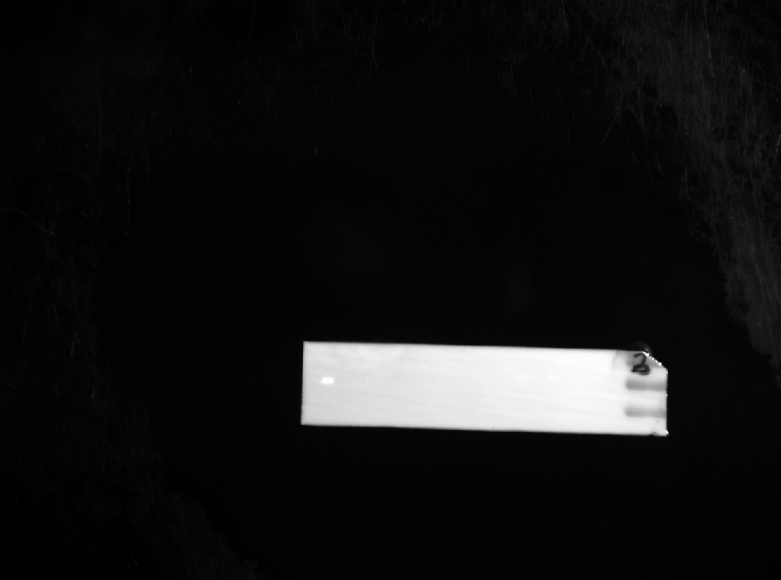

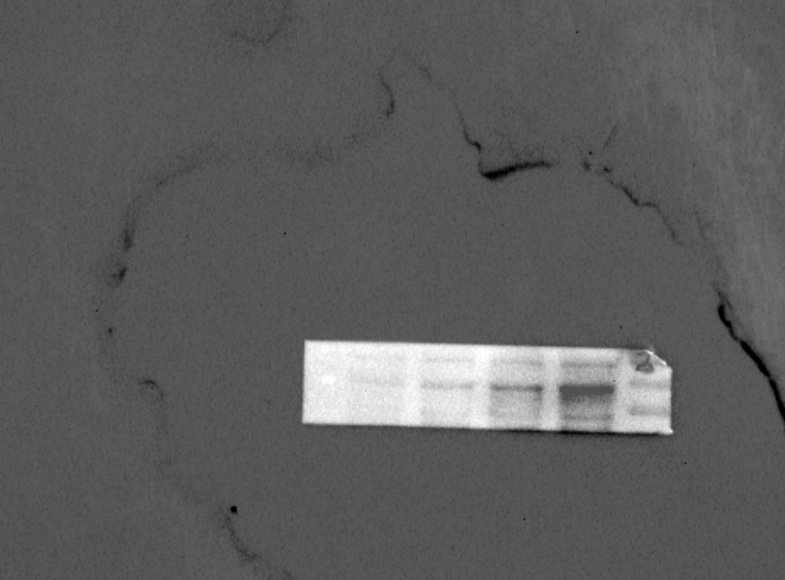


Figure 5. B The P62 protein band of H1299 cells.


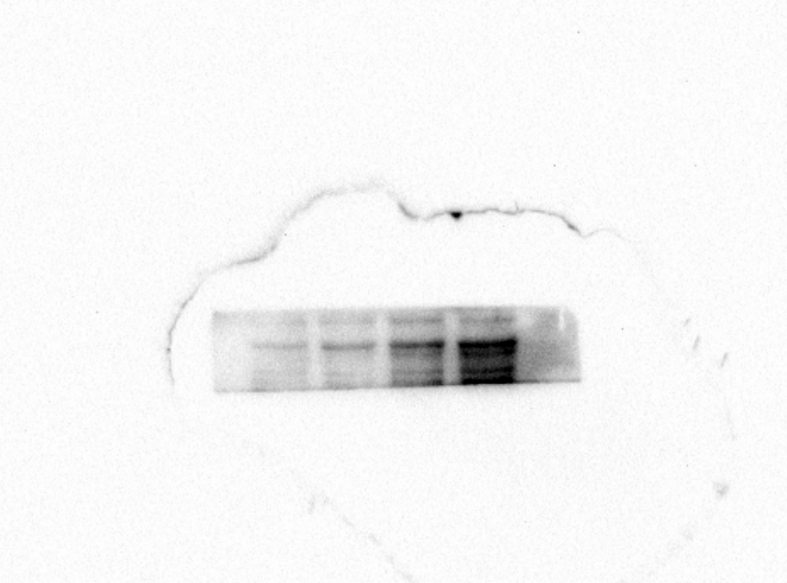

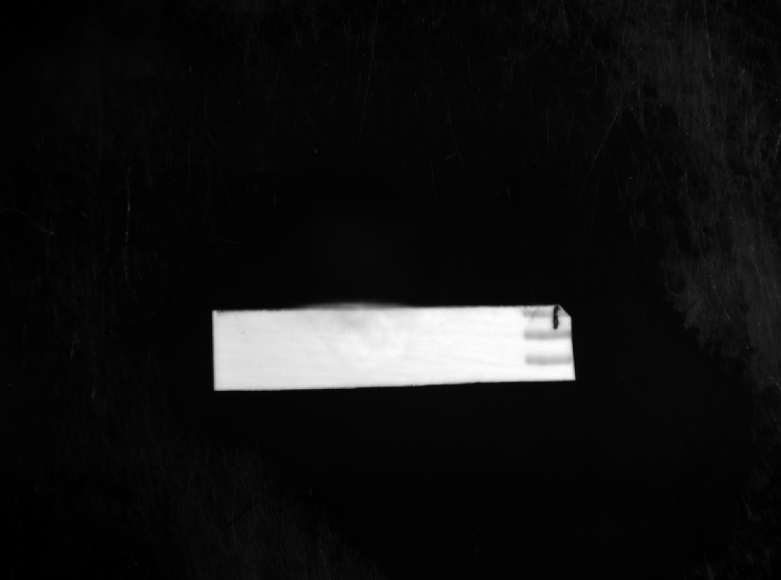

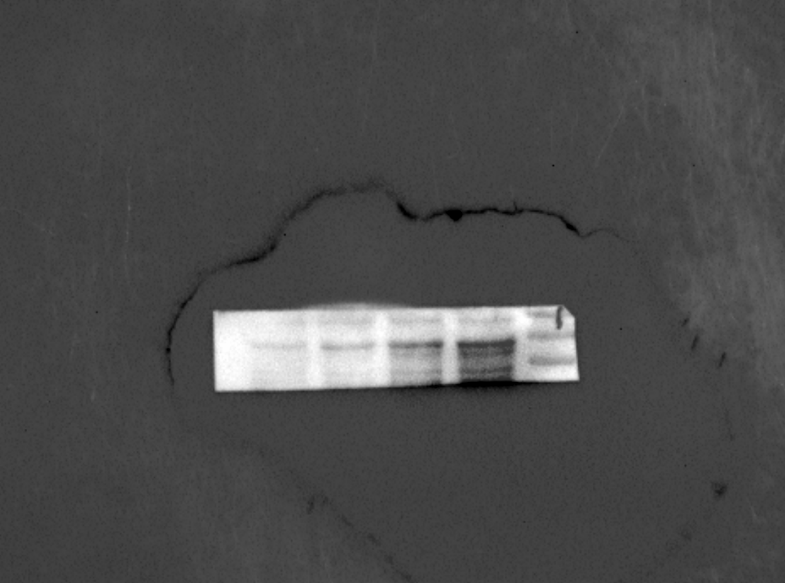


Figure 5. B The P62 protein band of A549 cells.


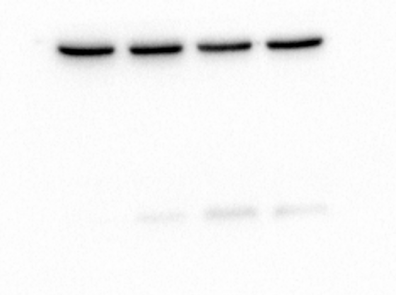

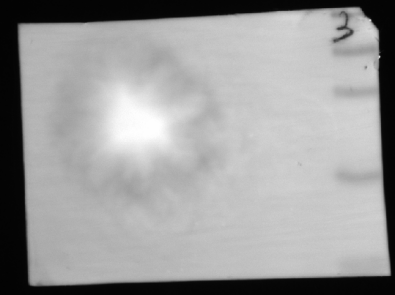

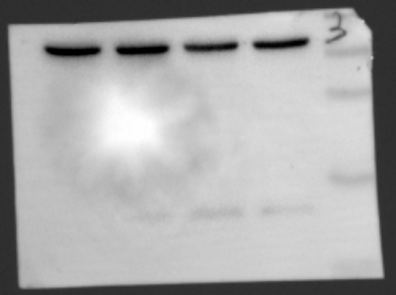


Figure5.B The GAPDH protein band of H1299 cells .


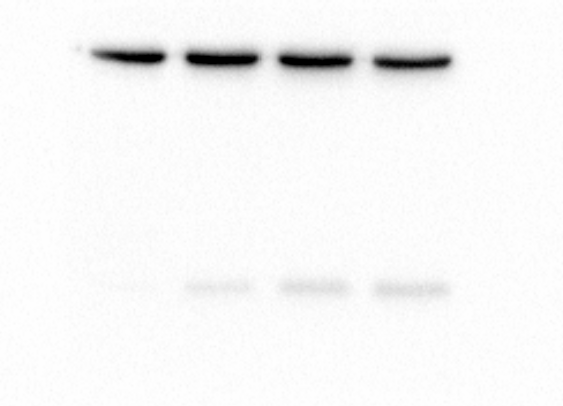

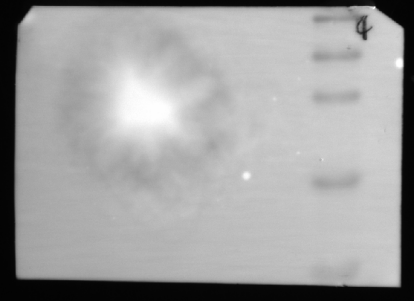

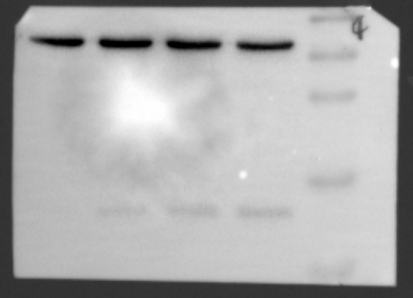


Figure5.B The GAPDH protein band of A549 cells .


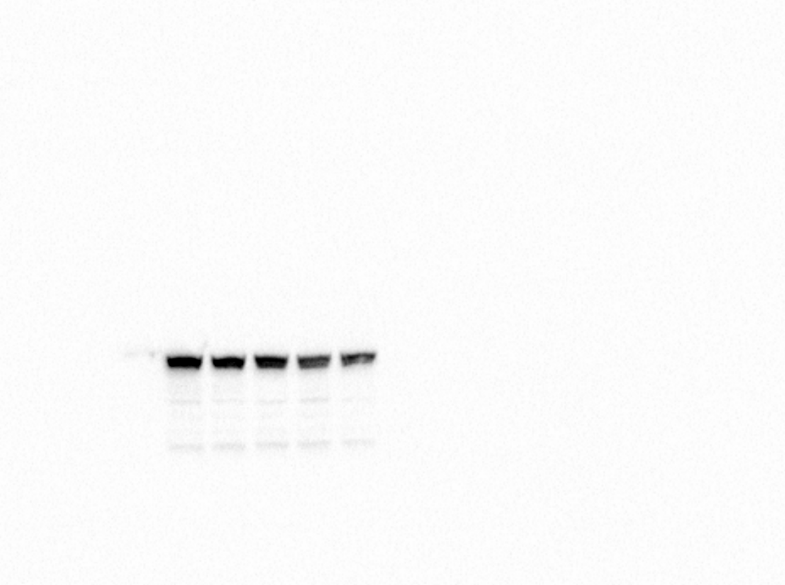

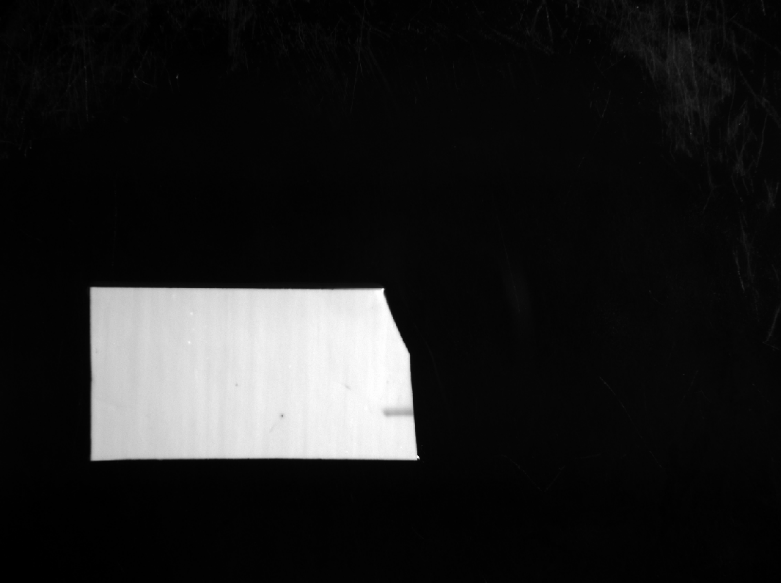

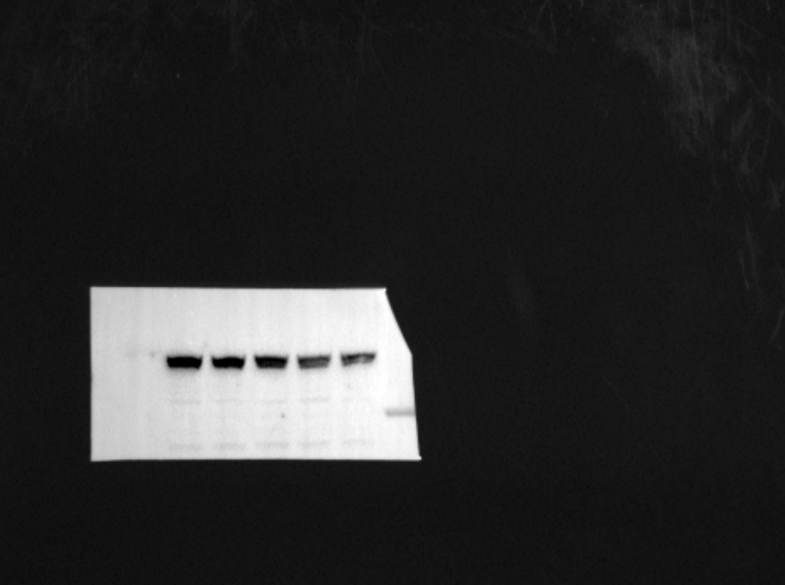


Figure5.C The mTOR protein band of H1299 cells .


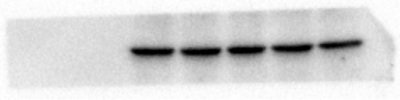

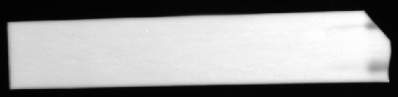

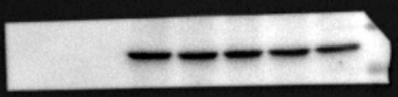


Figure5.C The GAPDH protein band of H1299 cells .


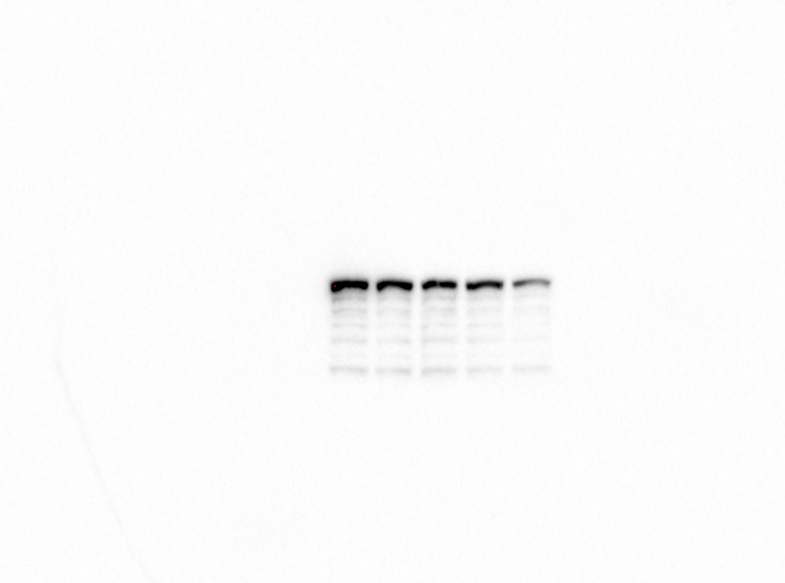

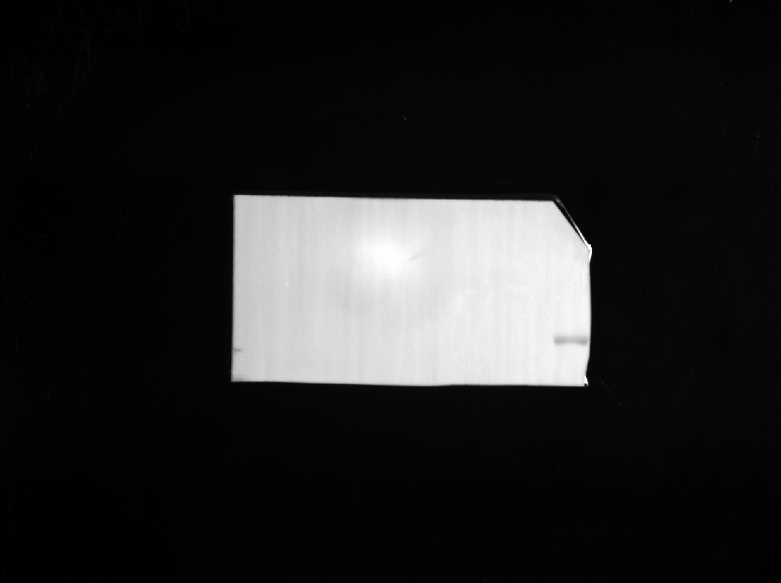

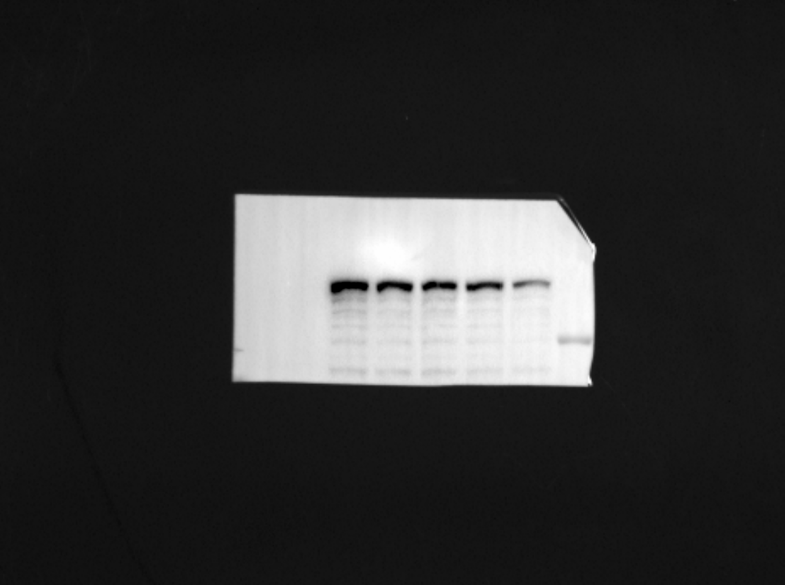


Figure5.C The mTOR protein band of A549 cells .


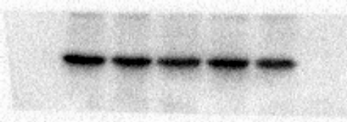

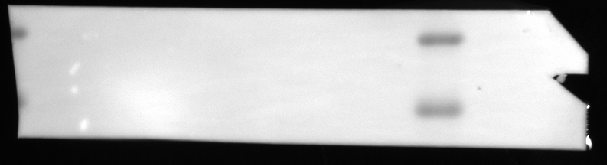

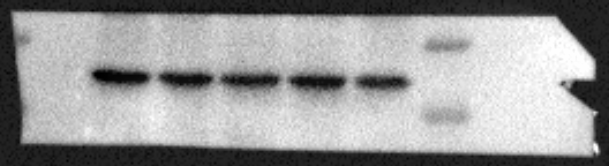


Figure5.C The GAPDH protein band of A549 cells .


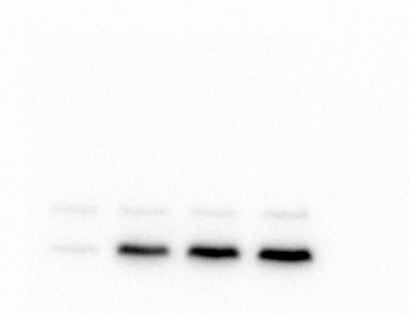

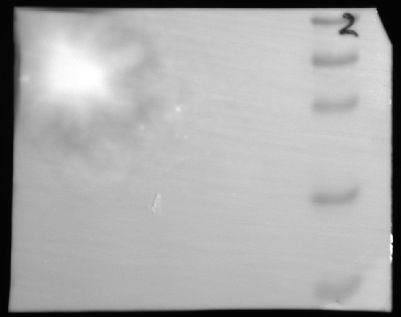

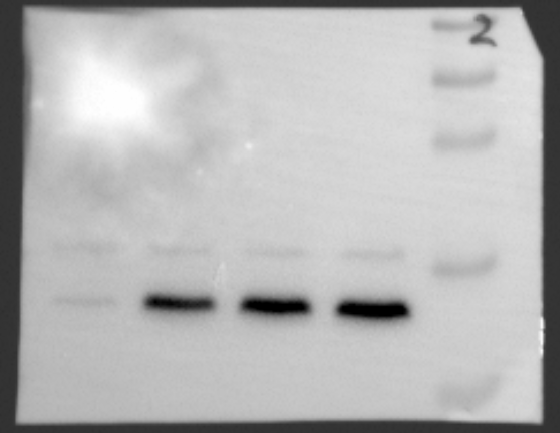


Figure6.C The LC3 protein band of H1299 cells .


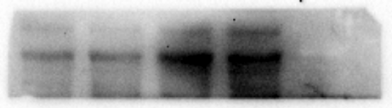

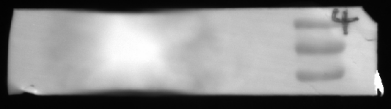

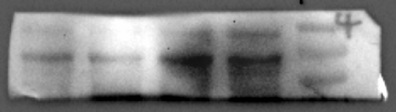


Figure6.C The P62 protein band of H1299 cells .


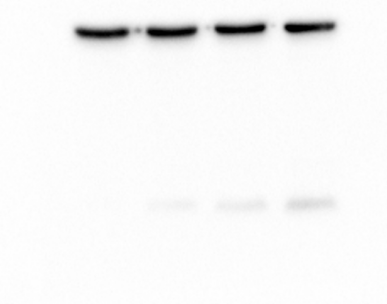

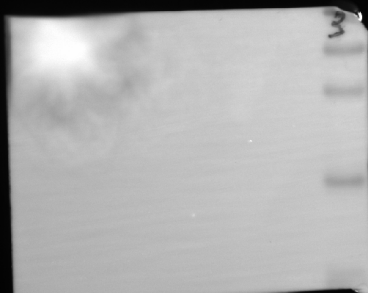

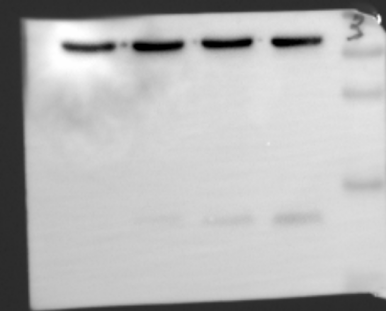


Figure6.C The GAPDH protein band of H1299 cells .


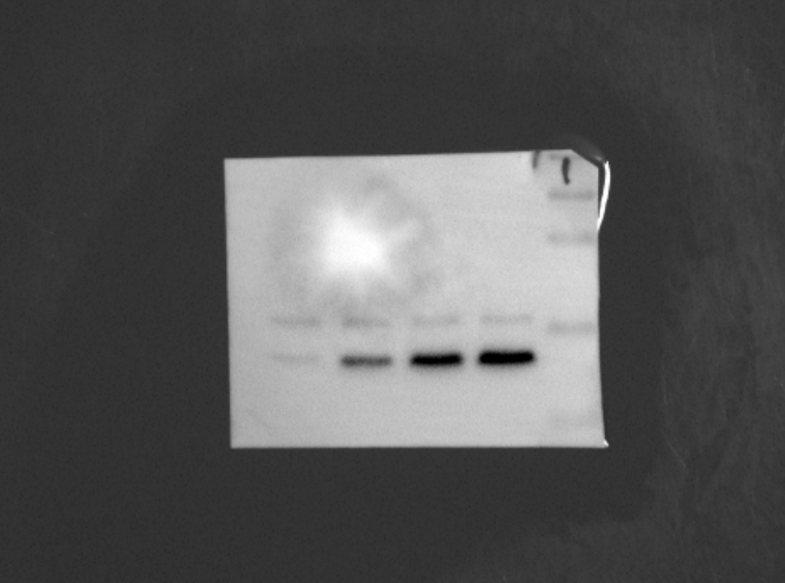

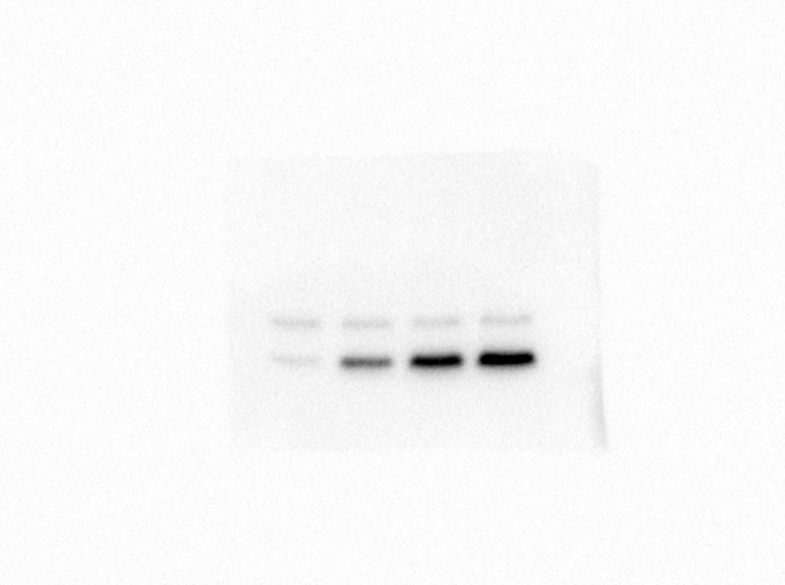

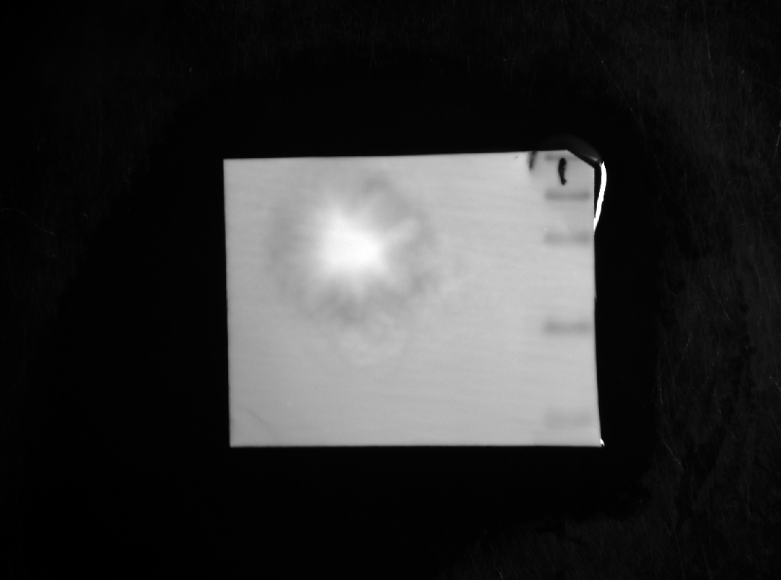


Figure6.D The LC3 protein band of a549 cells .


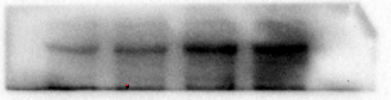

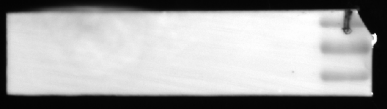

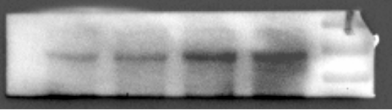


Figure6.D The P62 protein band of A549 cells .


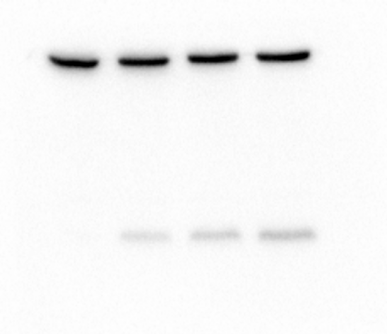

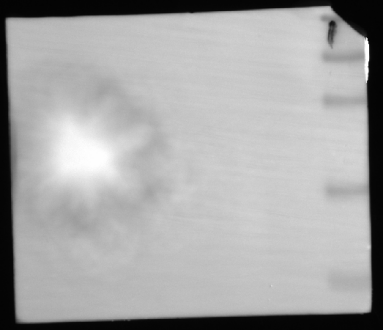

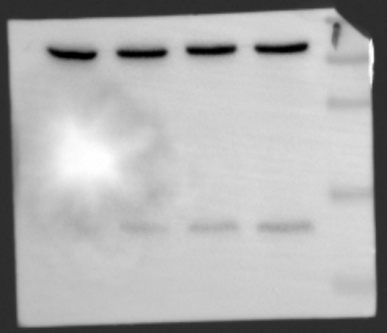


Figure6.D The GAPDH protein band of A549 cells .
